# Comparison of anesthesia methods for intra-arterial therapy of patients with acute ischemic stroke: an updated meta-analysis and systematic review

**DOI:** 10.1186/s12871-024-02633-3

**Published:** 2024-07-18

**Authors:** Huijun Chen, Yang Xing, Zekun Lang, Lei Zhang, Mao Liao, Ximin He

**Affiliations:** 1 Dingxi People’s Hospital, Dingxi, Gansu 743000 China; 2 The First Clinical Medical College of Lanzhou University, Lanzhou, Gansu 730000 China; 3 Department of Anesthesia and Surgery, First Hospital of Lanzhou University, Lanzhou, Gansu 730000 China; 4 The Second Clinical Medical College of Lanzhou University, Lanzhou, Gansu 730000 China

**Keywords:** Stroke, Intra-arterial therapy, General anesthesia, Local anesthesia, Conscious sedation

## Abstract

**Objectives:**

Currently, there remains debate regarding the optimal anesthesia approach for patients undergoing intra-arterial therapy for acute ischemic stroke. Therefore, we conducted a comparative analysis to assess the effects of general anesthesia versus non general anesthesia on patient outcomes.

**Methods:**

The research methodology entailed comprehensive searches of prominent databases such as the Cochrane Library, PubMed, Scopus, and Web of Science, covering the period from January 1, 2010, to March 1, 2024. Data synthesis employed techniques like risk ratio or standardized mean difference, along with 95% confidence intervals. The study protocol was prospectively registered with PROSPERO (CRD42024523079).

**Results:**

A total of 27 trials and 12,875 patients were included in this study. The findings indicated that opting for non-general anesthesia significantly decreased the risk of in-hospital mortality (RR, 1.98; 95% CI: 1.50 to 2.61; *p*<0.00001; I^2^ = 20%), as well as mortality within three months post-procedure (RR, 1.24; 95% CI: 1.15 to 1.34; *p*<0.00001; I^2^ = 26%), while also leading to a shorter hospitalization duration (SMD, 0.24; 95% CI: 0.15 to 0.33; *p*<0.00001; I^2^ = 44%).

**Conclusion:**

Ischemic stroke patients who undergo intra-arterial treatment without general anesthesia have a lower risk of postoperative adverse events and less short-term neurological damage. In routine and non-emergency situations, non-general anesthetic options may be more suitable for intra-arterial treatment, offering greater benefits to patients. In addition to this, the neuroprotective effects of anesthetic drugs should be considered more preoperatively and postoperatively.

**Supplementary Information:**

The online version contains supplementary material available at 10.1186/s12871-024-02633-3.

## Introduction

Stroke is a globally prevalent disease marked by high mortality and disability rates. It is classified into two main types based on its pathological features: ischemic stroke and hemorrhagic stroke. Ischemic stroke is the most common type of stroke, accounting for approximately 70% of all strokes [1]. The key to the treatment of acute ischemic stroke is to open the blocked blood vessels as early as possible and save the ischemic penumbra. The treatment method for early vascular recanalization of acute ischemic stroke that has been used for a long time in the past is mainly intravenous recombinant tissue plasminogen activator (rt-PA) thrombolysis [2–4]. Some studies have shown that intravenous rt-PA thrombolysis within 4.5 h of onset has clear benefits, and the earlier the thrombolysis, the greater the benefit [5]. However, intravenous thrombolysis has a strict time window limit, and the number of patients who can benefit from it is less than 3% of ischemic stroke patients. At the same time, there is still huge room for optimization of its therapeutic effect. Therefore, scholars around the world are exploring the intra-arterial therapy (IAT) of ischemic stroke [6]. Studies have shown that IAT based on mechanical thrombectomy can bring clear benefits and has now become the standard treatment for acute ischemic stroke in addition to intravenous thrombolysis [7–9].

As technology and materials improve, new problems arise. So far, the optimal anesthesia regimen for IAT in acute ischemic stroke remains controversial. General anesthesia (GA), as a widely-used method, offers several benefits. It effectively immobilizes the patient, minimizing involuntary movements. Additionally, it mitigates the risk of aspiration by managing the airway effectively, and it allows for superior control over circulation. Local anesthesia (LA) is also widely used in neurology-related surgeries. With the introduction of the concept of comfortable medicine, simple LA at the puncture point is no longer used, and is replaced by conscious sedation (CS) and monitored anesthesia care (MAC). CS has the characteristics of rapid onset of action and short preoperative preparation time, while MAC can better monitor hemodynamics and other vital signs of the patient to ensure safety. A retrospective study found that the use of GA during intra-arterial therapy had a more pronounced adverse effect on clinical outcomes than CS [10]. The results of several randomized controlled trials (RCTs) show that there is no significant difference in clinical outcomes between CS or GA for intra-arterial therapy [11–13]. Another meta-analysis showed that patients with anterior circulation stroke treated under GA may have better clinical neurological outcomes [14]. There is still controversy over which anesthesia regimen is best for intra-arterial therapy. Therefore, we collected relevant articles in recent years to conduct an updated meta-analysis and provide new guidance for clinical practice.

## Methods

The research adhered to the guidelines outlined in the Preferred Reporting Items for Systematic Reviews and Meta-Analyses Protocols (PRISMA) statement [15]. Furthermore, the protocol has been officially registered in the International Prospective Systematic Reviews Registry database with the registration number CRD42024523079.

### Sources of data and search strategy

A comprehensive search was carried out across several databases including PubMed, Web of Science, Scopus, and the Cochrane Library, spanning from January 2010 to March 2024, to identify studies related to acute ischemic stroke and anesthesia. Part of the search strategy is as follows: [(Stroke) OR (Cerebrovascular Accident)] AND [(Anesthesia, General) OR (Monitored anesthesia care) OR (Conscious Sedation)] in title/abstract. Furthermore, citations from articles were extracted to pinpoint relevant studies that might not have been initially captured during the literature search. The detailed search strategy is outlined in a Word document included within the supplementary materials.

### Inclusion and exclusion criteria

The inclusion criteria were established in accordance with the PICOS approach. These criteria include: (1) Original clinical studies contrasting GA with non-GA; (2) Participants aged over 18 years undergoing intra-arterial therapy, with baseline data and comorbidities not significantly special or high-risk; (3) Inclusion of pertinent clinical outcomes essential for this investigation. The exclusion criteria are as follows: (1) Literature types not classified as clinical trials, such as reviews, letters, and conference abstracts; (2) Studies lacking a comparison between GA and non-GA in intra-arterial therapy; (3) Insufficient data or inability to transform data into a usable format. Two authors independently reviewed and selected studies based on these predetermined criteria. Any discrepancies were resolved through discussion with a third party.

### Data collection and quality assessment

Two reviewers autonomously performed data extraction. Any disparities were resolved through consensus or by seeking input from a third party. The extracted data encompassed various details, including the primary author, publication year, sample size, participant demographics (age, gender), comorbidities, National Institutes of Health Stroke Scaleas (NIHSS) score, as well as primary and secondary outcomes.

In accordance with the “Randomized Trial Bias Risk Assessment Tool” as outlined in the Cochrane Handbook, the quality assessment of the randomized controlled trials (RCTs) encompasses several domains. These encompass allocation concealment, randomization method, blinding procedures for both investigators and participants, blinding of outcome assessors, selective reporting, completeness of data, and identification of other potential biases. The overall risk of bias assessment can lead to categorizations of low, unclear, or high risk of bias [16].

For retrospective studies, quality assessment was carried out utilizing the Newcastle-Ottawa Scale (NOS) by two independent reviewers. The assessment entailed evaluating three key aspects: selection bias, comparability, and exposure. Each aspect featured specific evaluation criteria, with stars allocated accordingly. The highest score attainable for comparability is two stars.

### Outcomes and definitions

The primary outcomes included functional independence at 3 months, in-hospital mortality, and mortality at 3 months. Functional independence at 3 months is defined as achieving an modified Rankin Scale (mRS) score of 0 to 2. Secondary outcomes were successful reperfusion, intracranial hemorrhage, pneumonia, NIHSS score after 24 h, vascular perforation, progressive ischemic stroke, and length of hospital stay. Successful reperfusion was defined as an modified Thrombolysis in Cerebral Infarction (mTICI) score of 2b or 3 indicating reperfusion of more than 50% of the affected area.

### Statistical analysis

All data underwent analysis using Review Manager (RevMan) version 5.4 (The Cochrane Collaboration, Copenhagen, Denmark) and Stata SE 16.0 (Stata Corporation, College Station, TX, USA). For dichotomous data, risk ratios (RR) with corresponding 95% confidence intervals (CI) were computed, while for continuous data, standard mean differences (SMD) with 95% CI were estimated. Both fixed and random effects models were employed to accommodate methodological and clinical heterogeneity. Heterogeneity among studies was evaluated using the Q-test and I^2^ statistic, with significant heterogeneity defined as *p* < 0.1 or I^2^ > 50%. Subgroup and meta-regression analyses were conducted to explore potential sources of heterogeneity. Publication bias was assessed through funnel plots, with Egger’s test employed when at least 10 studies were included. TSA 0.9.5.10 beta software was used to conduct trial sequential analysis (TSA) of clinical efficacy to reduce the occurrence of random errors, determine the reliability of the conclusions, and estimate the sample size required for meta-analysis. A significance level of α = 0.05 was utilized for all analyses. Sensitivity analysis was conducted to assess result robustness and to identify potential sources of heterogeneity.

## Results

### Literature selection

**Fig. 1:**
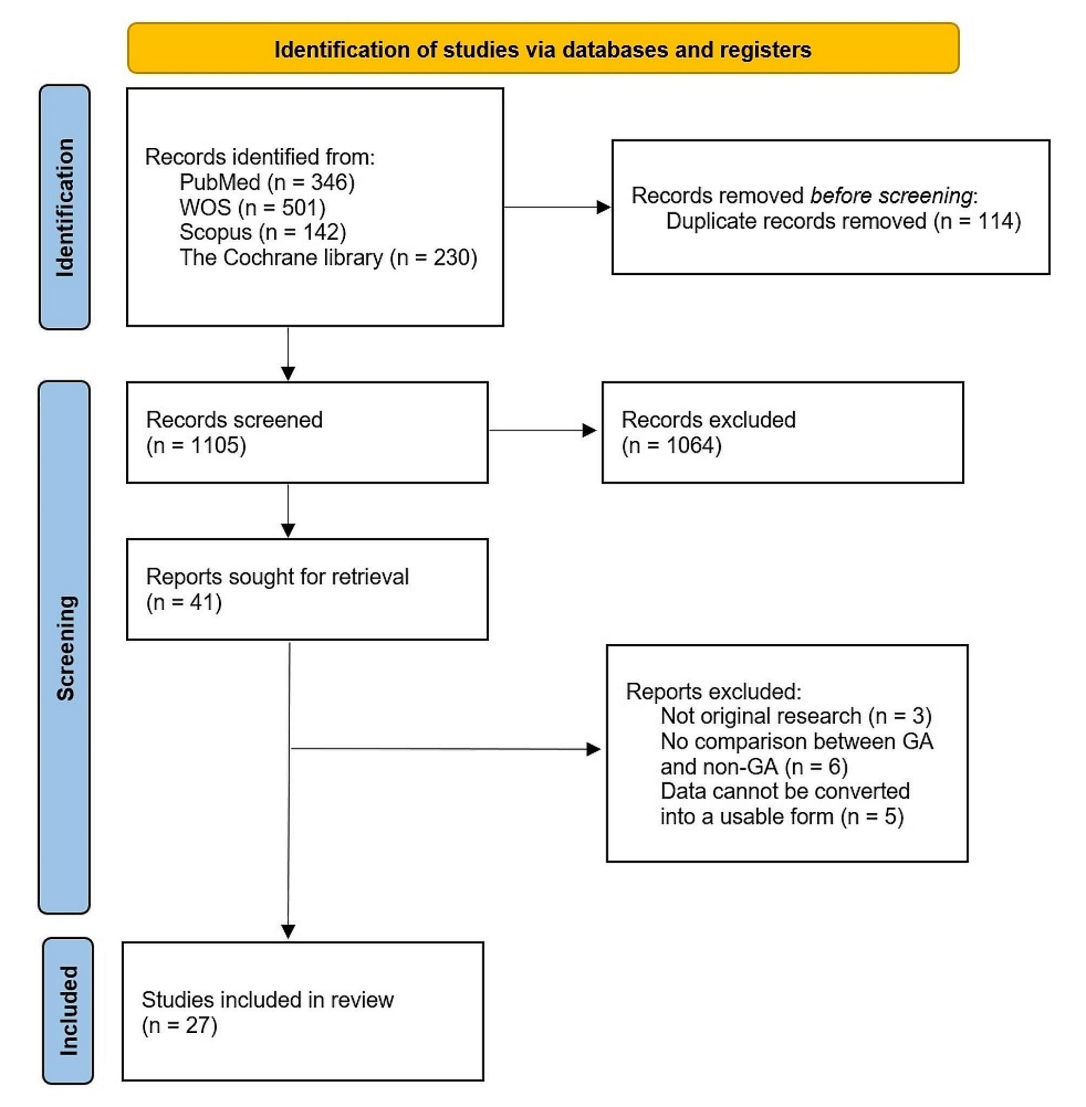
Preferred reporting items for systematic reviews and meta-analyses (PRISMA) flowchart of selection

A total of 1219 pieces of literature were identified across various databases. Following the removal of 114 duplicate studies, a preliminary screening excluded 1105 studies. Subsequently, 41 articles underwent full-text evaluation, ultimately resulting in the inclusion of 27 trials for final analysis. Within this selection, three articles were omitted as they did not constitute original clinical studies, seven articles were disregarded due to the absence of a comparison between general anesthesia (GA) and non-GA, and five articles were excluded either due to the lack of relevant results or the inability to convert the data into a usable format. The specific screening process is detailed in Fig. 1. Among the 27 included articles, 12 were RCTs and 15 were cohort studies [11, 12, 17–41].

### Baseline characteristic and quality assessment

The trials analyzed in this study were all published post-2010, featuring sample sizes ranging from 40 to 4429 individuals. In total, 12,875 participants were included, with an average age of 69.5 years. The baseline demographic characteristics and comorbidities of the patients are detailed in Table 1.

**Table 1:** Basic information included in the studies

Reference	Sample size	Age	Male	HT	AF	DM	HL	Smoking	CAD	HF	NIHSS
Abou [17]	281	67.2 ± 15.0	145 (51.6)	211 (75.1)	112 (39.9)	72 (25.6)	138 (49.1)	87 (31.0)	85 (30.2)	NA	18.1 ± 6.6
Bekelis [18]	1174	67.3 ± 15.0	559 (47.6)	791 (67.4)	NA	289 (24.6)	475 (40.5)	151 (12.9)	322 (27.4)	313 (26.7)	NA
Berkhemer [19]	216	65.0 ± 16.4	126 (58.3)	NA	62 (28.7)	31 (14.4)	NA	NA	NA	NA	17.6 ± 4.9
Cappellari [20]	4429	71.1 ± 14.0	2241 (50.6)	2442 (55.1)	1155 (26.1)	641 (14.5)	511 (11.5)	785 (17.7)	382 (8.6)	267 (6.0)	17.6 ± 5.4
Chabanne [21]	273	71.6 ± 13.8	131 (48.0)	167 (61.2)	NA	38 (13.9)	NA	NA	NA	29 (10.6)	15.5 ± 6.7
Farag [22]	358	67.7 ± 15.0	174 (48.6)	323 (90.2)	NA	133 (37.2)	86 (24.0)	NA	NA	103 (28.8)	15.2 ± 7.3
Goldhoorn [23]	1376	69.5 ± 15.0	742 (53.9)	696 (50.6)	308 (22.3)	231 (16.8)	414 (30.1)	313 (22.7)	NA	NA	15.6 ± 6.4
Hu [24]	139	72.0 ± 7.1	72 (51.8)	65 (46.8)	51 (36.7)	21 (15.1)	49 (35.3)	40 (28.8)	NA	NA	NA
Jagani [25]	99	66.1 ± 12.4	52 (52.5)	75 (75.8)	31 (31.3)	17 (17.2)	NA	46 (46.5)	27 (27.3)	NA	NA
Janssen [26]	84	69.8 ± 12.5	38 (45.2)	64 (76.2)	42 (50.0)	10 (11.9)	22 (26.2)	21 (25.0)	NA	NA	NA
John [27]	190	67.0 ± 15.2	83 (43.7)	137 (72.1)	75 (39.5)	45 (23.7)	85 (44.7)	NA	NA	NA	15.8 ± 6.6
Just [28]	109	61.9	67 (35.3)	61 (56.0)	NA	19 (17.4)	NA	52 (47.7)	NA	NA	13.1
Li [29]	109	66.1 ± 16.3	53 (48.6)	79 (72.5)	32 (29.4)	27 (24.8)	51 (46.8)	NA	38 (34.9)	NA	16.0 ± 6.3
Li [30]	636	NA	359 (56.4)	380 (59.7)	294 (46.2)	120 (18.9)	NA	NA	NA	NA	NA
Liang [31]	87	62.0 ± 12.0	71 (81.6)	63 (72.4)	14 (16.1)	23 (26.4)	31 (35.6)	53 (60.9)	13 (14.9)	NA	15.7 ± 5.7
Maurice [32]	351	71.7 ± 12.6	194 (55.3)	221 (63.0)	107 (30.5)	49 (14.0)	NA	NA	NA	NA	16.0 ± 5.5
Mundiyanapurath [33]	44	72.3 ± 14.1	19 (43.2)	NA	NA	NA	NA	NA	NA	NA	19.2 ± 7.1
Peng [34]	149	63.5 ± 12.9	92 (61.7)	84 (56.4)	60 (40.3)	14 (9.4)	7 (4.7)	43 (28.9)	NA	NA	16.0 ± 5.9
Pop [35]	361	73.0 ± 15.1	169 (46.8)	242 (67.0)	NA	70 (19.4)	123 (34.1)	57 (15.8)	NA	NA	15.2 ± 7.2
Ren [36]	90	69.2 ± 6.1	50 (55.6)	37 (41.1)	9 (10.0)	11 (12.2)	6 (6.7)	NA	NA	NA	13.6 ± 3.8
Schonenberger 2016	150	71.5 ± 13.8	90 (60.0)	107 (71.3)	72 (48.0)	34 (22.7)	44 (29.3)	22 (14.7)	NA	38 (25.3)	17.0 ± 3.8
Simonsen [12]	128	71.4 ± 11.4	66 (51.6)	71 (55.5)	51 (39.8)	18 (14.1)	NA	40 (31.3)	NA	NA	17.5 ± 5.4
Sun [38]	40	63.2 ± 19.2	26 (65.0)	17 (42.5)	12 (30.0)	9 (22.5)	NA	NA	7 (17.5)	NA	13.7 ± 6.0
Vandenberg 2015	348	61.3 ± 14.7	184 (52.9)	167 (48.0)	90 (25.9)	49 (14.1)	85 (24.4)	NA	NA	NA	NA
Wagner [39]	1284	71.6 ± 13.6	667 (51.9)	895 (69.7)	512 (39.9)	222 (17.3)	819 (63.8)	270 (21.0)	NA	NA	14.0 ± 7.7
Wu [41]	187	64.1 ± 10.9	127 (67.9)	97 (51.9)	53 (28.3)	46 (24.6)	39 (20.9)	79 (42.2)	NA	NA	14.3 ± 6.6
Wu [40]	183	59.7 ± 11.6	148 (80.9)	141 (77.0)	31 (16.9)	54 (29.5)	31 (16.9)	87 (47.5)	NA	NA	22.0 ± 14.5

HT, Hypertension; AF, Atrial fibrillation; DM, Diabetes mellitus; HL, Hyperlipidemia; CAD, Coronary artery disease; HF, heart failure; NIHSS, National Institutes of Health

Stroke Scale; NA, not applicable

Data are expressed as mean ± SD or mean or n (%)

After a thorough quality assessment of twelve randomized controlled trials, two were determined to have a high risk of bias, five to have a low risk of bias, and the remaining five were considered to have an unclear risk of bias (Figure S1). After assessing the quality of the remaining fifteen retrospective studies, we found that all studies had above-average NOS scores. Each study scored more than five stars and met the criteria for inclusion in the meta-analysis. The conclusive results are detailed in Table S1.

### Main outcomes

Non-GA is associated with a smaller risk of in-hospital death (RR, 1.98; 95% CI: 1.50 to 2.61; *p*<0.00001; I^2^ = 20%) and three-month mortality (RR, 1.24; 95% CI: 1.15 to 1.34; *p*<0.00001; I^2^ = 26%) than GA. Non-GA is associated with higher successful reperfusion (RR, 1.06 ; 95% CI: 1.01 to 1.11; *p* = 0.02; I^2^ = 60%) and lower risk of progressive ischemic stroke (RR, 1.41 ; 95% CI: 1.10 to 1.79 ; *p* = 0.006; I^2^ = 27%). In addition, patients without GA had lower NIHSS scores 24 h after surgery (SMD, 0.13; 95% CI: 0.01 to 0.25; *p* = 0.03; I^2^ = 54%), but the mRS score results at three months (RR, 0.89 ; 95% CI: 0.79 to 0.99 ; *p* = 0.04; I^2^ = 55%) showed that patients with GA had better outcomes. Patients without GA had shorter hospital stays (SMD, 0.24; 95% CI: 0.15 to 0.33; *p*<0.00001; I^2^ = 44%).There may be a lower risk of vascular perforation and a higher risk of intracranial hemorrhage after general anesthesia, but this result is not statistically significant, and there is no significant difference in the risk of postoperative pneumonia. Relevant results are summarized in Tables 2 and 3, and forest plots are shown in the supplementary material.

**Table 2:** Summary of main meta-analysis results

Outcomes	No. of studies	Participants	Statistical Method	Effect Estimate	I^2^	*p*-value
mRS ≤ 2 after three months						
RCT	8	2927	RR (M-H, Random)	0.94 [0.79, 1.11]	53%	0.45
Non-RCT	10	6378	RR (M-H, Random)	0.84 [0.71, 1.00]	58%	0.05
Total	18	9305	RR (M-H, Random)	0.89 [0.79, 0.99]	55%	0.04
Three-months mortality						
RCT	9	2644	RR (M-H, Fixed)	1.02 [0.87, 1.18]	0	0.84
Non-RCT	10	8288	RR (M-H, Fixed)	1.32 [1.21, 1.44]	24%	<0.00001
Total	18	10,932	RR (M-H, Fixed)	1.24 [1.15, 1.34]	26%	<0.00001
In-hospital death						
RCT	3	327	RR (M-H, Fixed)	1.04 [0.53, 2.04]	0	0.90
Non-RCT	4	766	RR (M-H, Fixed)	2.33 [1.71, 3.19]	0	<0.00001
Total	7	1093	RR (M-H, Fixed)	1.98 [1.50, 2.61]	20%	<0.00001
Successful reperfusion						
RCT	11	2164	RR (M-H, Fixed)	1.07 [1.02, 1.12]	36%	0.007
Non-RCT	10	6393	RR (M-H, Fixed)	1.01 [0.98, 1.04]	68%	0.36
Total	21	8557	RR (M-H, Random)	1.06 [1.01, 1.11]	60%	0.02
NIHSS score after 24 h						
Total (All RCT)	9	3319	SMD (IV, Random)	0.13 [0.01, 0.25]	54%	0.03
Progressive ischemic stroke						
RCT	3	1865	RR (M-H, Random)	1.40 [1.06, 1.86]	67%	0.02
Non-RCT	3	718	RR (M-H, Fixed)	1.42 [0.88, 2.29]	0	0.16
Total	6	2583	RR (M-H, Fixed)	1.41 [1.10, 1.79]	27%	0.006
Length of hospital stay						
RCT	5	2012	SMD (IV, Fixed)	0.10 [-0.05, 0.25]	19%	0.21
Non-RCT	2	699	SMD (IV, Fixed)	0.32 [0.21, 0.43]	0	<0.00001
Total	7	2012	SMD (IV, Fixed)	0.24 [0.15, 0.33]	44%	<0.00001

**Table 3:** Other outcomes and statistical results

Outcomes	Studies	Participants	Risk ratio	95% CI	*p*-value	Corresponding figure
Intracerebral hemorrhage	18	10,710	1.08	0.93–1.26	0.32	Figure S9
Pneumonia	11	3058	1.01	0.85–1.20	0.93	Figure S10
Vessel perforations	6	1299	0.51	0.24–1.08	0.08	Figure S11

### Subgroups analysis

We conducted subgroup analysis on mRS ≤ 2 after three months, three-month mortality, in-hospital death, successful reperfusion and length of hospital stay according to different study types. The trends reported in RCT studies and non-RCT studies are basically the same, and no significant difference. In addition, we found that different study types may be one of the sources of heterogeneity in three-month mortality, in-hospital death, successful reperfusion and length of hospital stay.

**Fig. 2:**
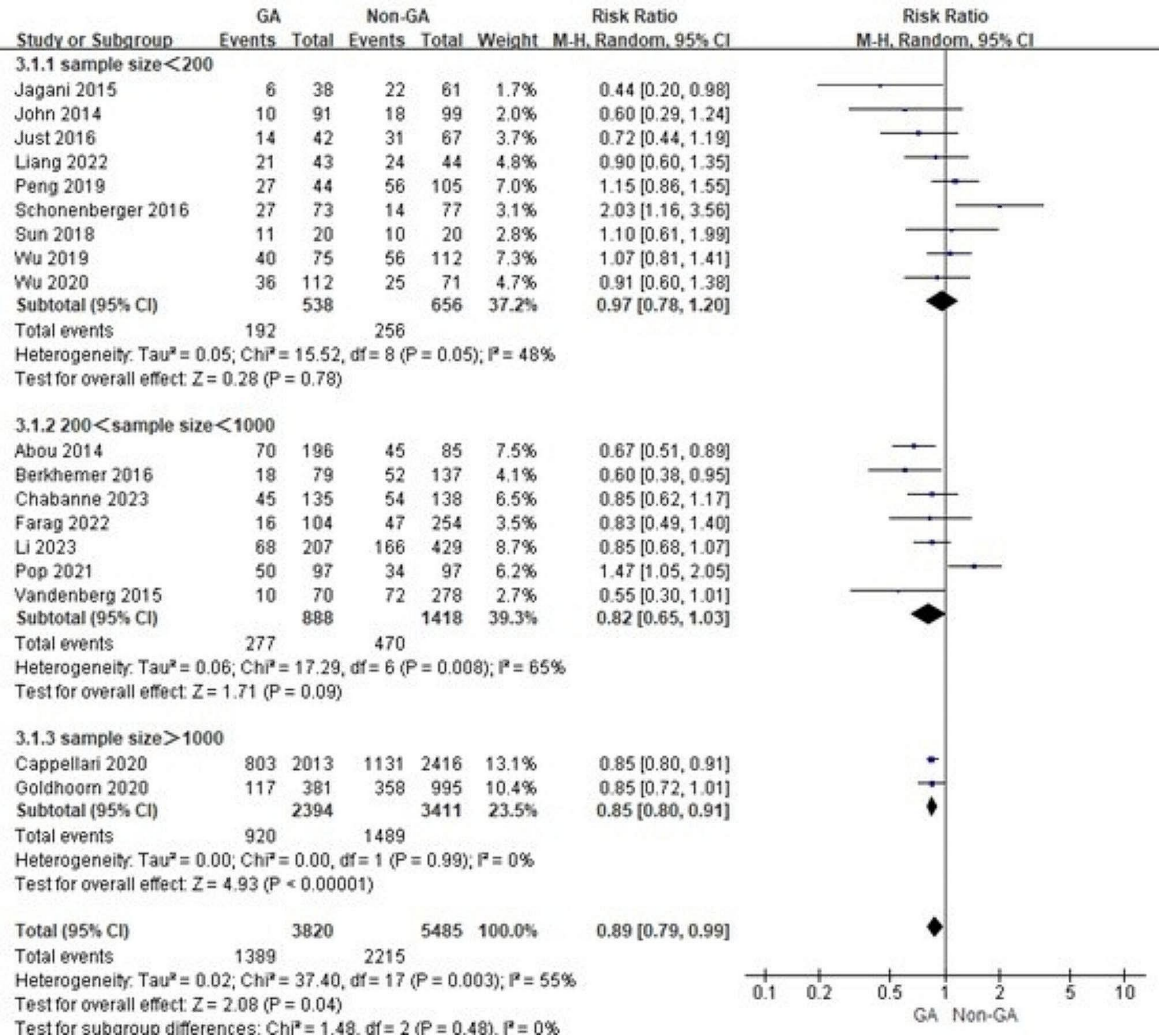
Subgroup analysis of mRS ≤ 2 after three months based on different sample size

**Fig. 3:**
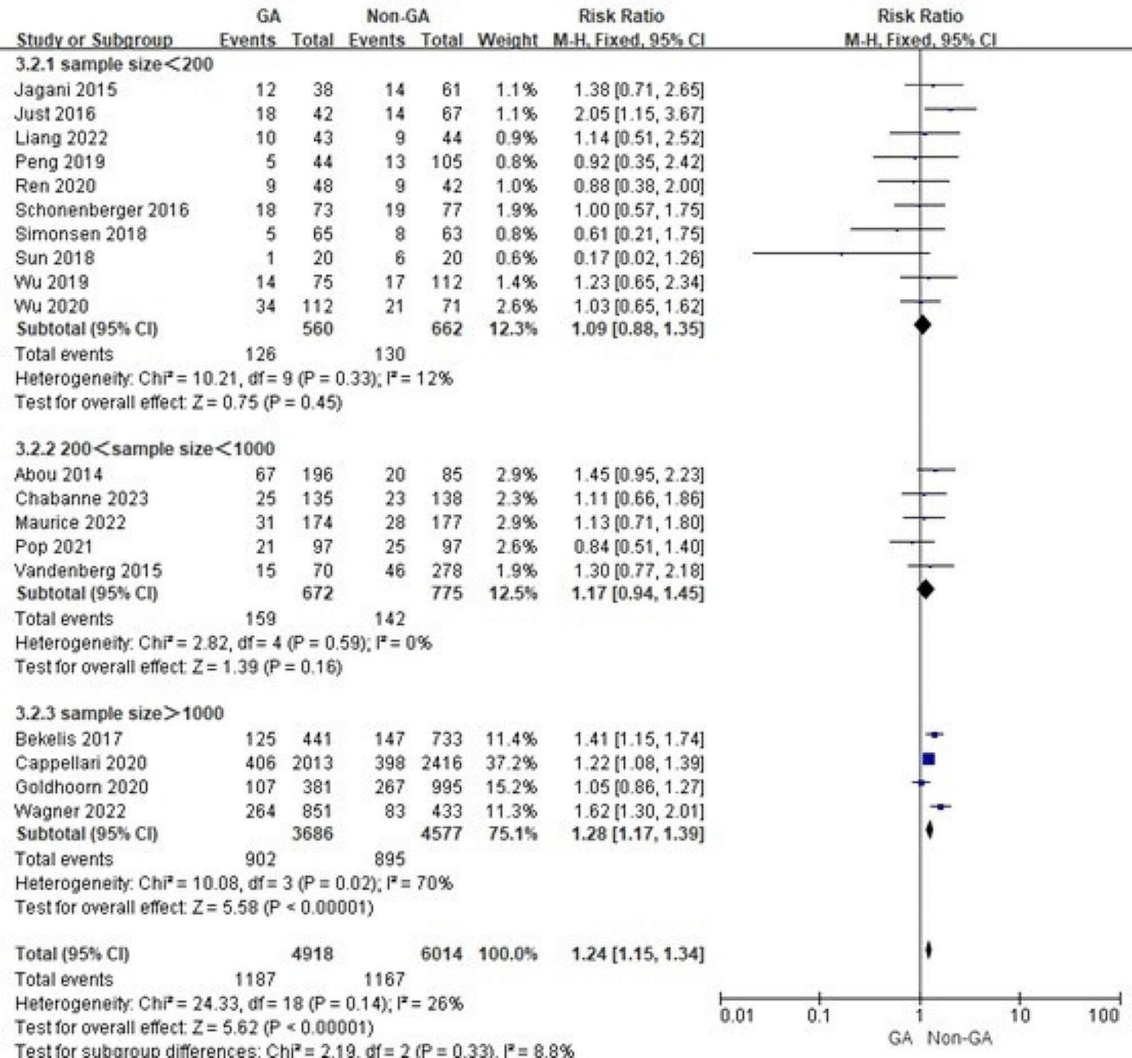
Subgroup analysis of three-months mortality based on different sample size

**Fig. 4:**
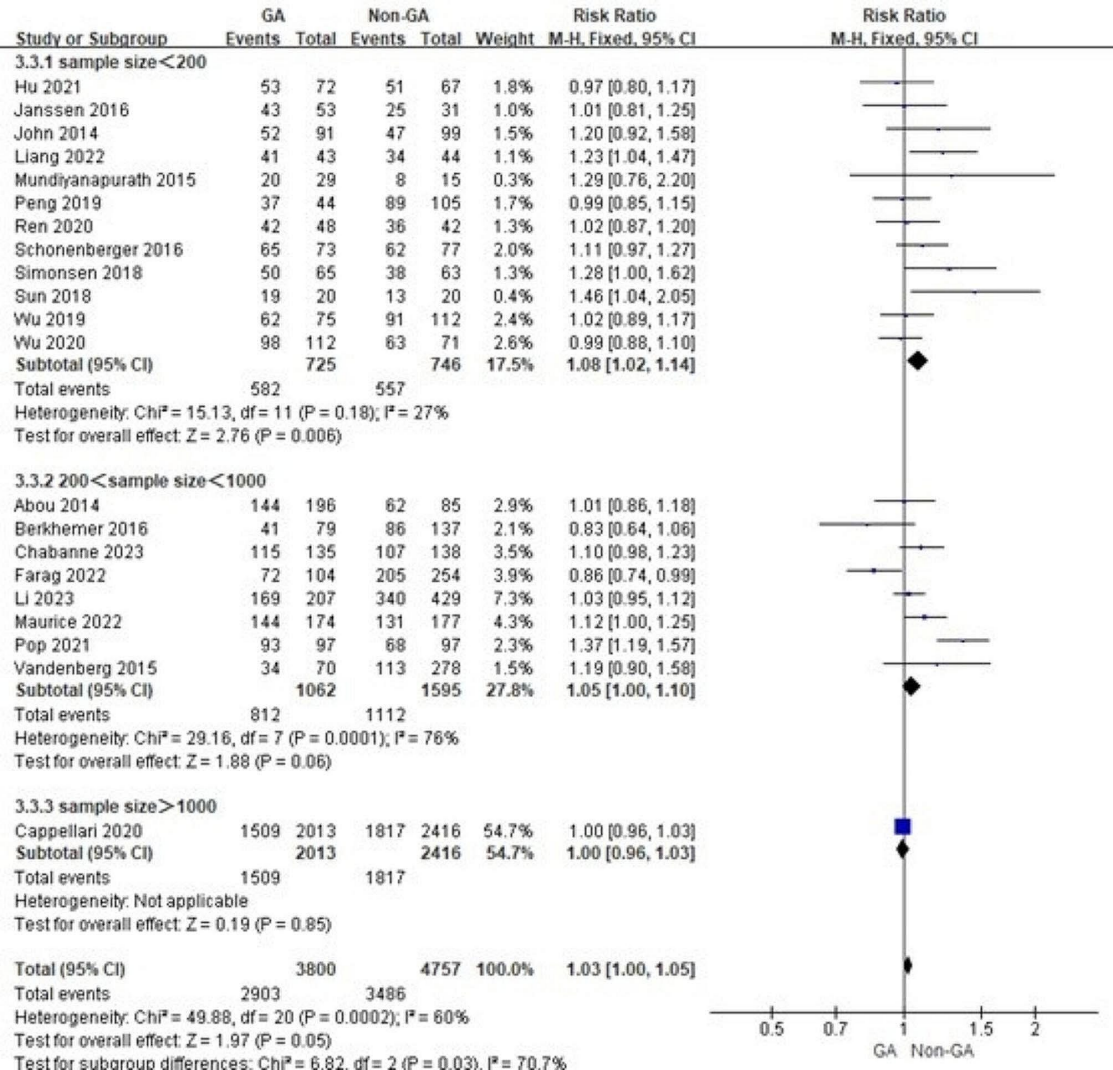
Subgroup analysis of recanalization success based on different sample size

Considering the relationship between surgical volume and outcomes for complex and high-risk surgeries, differences in sample size may have influenced the results. Therefore, we grouped mRS ≤ 2 after three months, three-months mortality and recanalization success according to different sample size levels and conducted subgroup analysis. The results show that differences in sample size do not significantly affect the results and that sample size is not a source of heterogeneity (Figs. 2, 3 and 4).

### Meta-regression

A random effects multivariable meta-regression analysis was performed to examine the association between mRS ≤ 2 three months post-stroke, NIHSS score at 24 h and successful reperfusion. Factors including age, gender, hypertension, hyperlipidemia, smoking, and NIHSS score were taken into account. None of these factors were identified as potential sources of heterogeneity. Detailed results are presented in the supplementary material. Considering the possible influence of sample size on the results, we also conducted a meta-regression with sample size as the covariate for mRS ≤ 2 after three months, three-months mortality and recanalization success. The results showed that sample size was not the source of heterogeneity.

### Publication bias and sensitivity analysis

The funnel plots for all findings revealed no substantial evidence of publication bias. Furthermore, both Egger’s and Begg’s tests were conducted, confirming the absence of publication bias across all outcomes. For outcomes with fewer than 10 included studies, publication bias was not examined. Detailed funnel plots and test outcomes are available in the supplementary materials. Additionally, the sensitivity analysis underscores the robustness of our results (accessible in the supplementary materials).

### TSA

This study conducted TSA for mRS ≤ 2 after three months and three-months mortality, setting the type I error rate α = 0.05, the information axis as the cumulative sample size, the statistical power of 80%, and the sample size as the required information size (RIS), see Figure S15 and Figure S16. As a result, the Z-curve crossed both the traditional boundary and the TSA boundary, and its cumulative information volume reached RIS. It shows that under the effect of RR = 0.89, non-GA has clear evidence for improving the three-month neurological prognosis of patients. With the effect of RR = 1.24, the evidence that GA can improve the three-month mortality rate of patients is conclusive.

## Discussion

The most effective anesthetic approach for IAT in ischemic stroke continues to be a subject of debate and contention among medical professionals. Current guidelines advise tailoring decisions to individual patient characteristics, yet they do not offer precise recommendations [3]. Previous studies have yielded varying conclusions regarding the advantages and disadvantages of different anesthesia methods for ischemic stroke patients undergoing IAT. Möhlenbruch et al [42].‘s study, comprising 111 patients who received IAT for posterior circulation stroke, found that patients under CS exhibited significantly lower mRS scores 90 days post-treatment compared to those under GA. Conversely, two other studies indicated that GA was associated with poorer functional outcomes at the three-month mark [43, 44]. However, some research suggests that anesthesia methods may not significantly correlate with clinical functional outcomes. For instance, Nogueira et al [45].‘s case-control study involving 215 patients with posterior circulation stroke who underwent IAT revealed similar rates of successful reperfusion, functional independence, hemorrhagic transformation, and mRS scores between the GA and CS groups. Similarly, Peng et al [46].‘s study, encompassing 639 patients with basilar artery occlusion undergoing IAT, found no statistically significant differences in favorable functional outcomes, mortality, hemorrhagic transformation, or three-month mRS scores among patients undergoing GA, LA or CS.

Our study encompassed a substantial volume of documents, with primary findings indicating notable benefits associated with non-GA compared to GA Notably, the GA cohort exhibited elevated risks of mortality, disease advancement, and prolonged hospitalization. Intriguingly, while the GA group demonstrated improved mRS scores at three months post-surgery compared to the non-GA group, their NIHSS scores 24 h after surgery were inferior. There was acceptable heterogeneity in some of our outcomes, and we also conducted subgroup analysis, meta-regression, and sensitivity analysis to explore heterogeneity. The results indicate that different study types may be one of the sources of heterogeneity in three-month mortality, in-hospital death, successful reperfusion and length of hospital stay. In addition, when excluding the study by Wagner et al [39], the I^2^ for three-month mortality dropped to 4%, *p* < 0.0001, and the RR was 1.19. When the study by Farag et al [22] is eliminated, the I^2^ of in-hospital death drops to 0, *p* = 0.001, and the RR is 1.69. Clinical heterogeneity caused by different treatment plans, anesthesia plans, and nursing plans adopted by different centers is also one of the sources of heterogeneity in this study. Overall, our heterogeneity is small and acceptable and does not affect the reliability of the results of this study.

Patients who do not receive GA may experience quicker neurological recovery within 24 h post-surgery, as indicated by lower NIHSS scores. Conversely, patients undergoing surgery with GA may enjoy more consistent and enduring treatment outcomes over the course of the three-month observation period, potentially resulting in improved performance on mRS scores. In the immediate aftermath of surgery, patients who did not receive GA demonstrated superior performance on NIHSS scores, potentially due to a prompt restoration of neurological function post-operation. Conversely, patients who underwent surgery under GA exhibited improved mRS scores several months later, suggesting they may have benefited from the neuroprotective properties of GA over an extended duration, facilitating a more favorable recovery trajectory. Such distinctions could arise from varying physiological and neurological responses at different stages following surgery [47]. An alternative perspective suggests that surgery under anesthesia offers enhanced intraoperative control and the capacity to address complications effectively, thereby facilitating superior long-term neurological recovery. Conversely, patients not under GA tended to experience swifter recovery in the immediate postoperative period, evident in their superior NIHSS scores within 24 h of surgery. This phenomenon could be partially attributed to the transient neurological depression induced by anesthetic drugs, although such effects might not endure over longer durations [48]. However, further research may be necessary to definitively ascertain the exact cause.

In addition, for high-risk procedures such as IAT in patients with acute ischemic stroke, the volume of surgery performed by a medical center may have a certain impact on the patient’s outcomes. A large number of studies have shown that there is a certain relationship between the volume of complex and high-risk surgeries and outcomes [49]. High-level medical centers may have more resources, more advanced equipment, and more experienced medical teams. Medical staff in low-sample centers may lack experience and training. This may have a certain impact on the results, so we also adopted subgroup analysis, meta-regression, and TSA to minimize this impact.

Propofol stands out as the predominant intravenous anesthetic in contemporary anesthesia practice. It mitigates post-ischemic neuronal damage through a variety of mechanisms, including the activation of GABAA receptors, exertion of antioxidant effects, reduction of brain mitochondrial membrane permeability, and augmentation of glutamate uptake. Additionally, Propofol diminishes cerebral blood flow (CBF), intracranial pressure, and cerebral oxygen metabolic rate, making it the preferred anesthetic for neurosurgical procedures [50]. Ketamine, an NMDA receptor antagonist, offers a distinct profile in that while it elevates CBF, its impact on overall cerebral oxygen metabolic rate remains minimal. Its neuroprotective properties stem from its ability to thwart the excitotoxic effects of glutamate, a mechanism supported by findings from in vitro and animal studies [51]. Moreover, intravenous lidocaine has emerged as another agent with demonstrated neuroprotective effects in the context of hypoxia-ischemia. This effect is likely attributed to its inhibition of sodium uptake and reduction of neuroinflammation, as evidenced by both in vitro and animal research [52]. In summary, the variance in prognosis observed among different anesthesia methods following IAT treatment for ischemic stroke may, in part, be attributed to the neuroprotective effects of anesthetic drugs. GA could potentially confer neuroprotective benefits in certain disease states linked to cerebral ischemia. While animal experiments offer robust support for this notion, clinical evidence remains scarce. Hence, future research should prioritize investigating the neuroprotective properties of various anesthetic drugs. Subsequently, these findings can inform the selection of safer medications tailored to specific clinical patients or scenarios. This approach aims to fulfill the demands for swift recovery and personalized diagnosis and treatment.

Although our study included a large number of existing studies, it still has the following limitations: First, half of the included literature were non-randomized controlled studies, which may put us at a disadvantage in terms of the level of evidence. Second, we were unable to conduct subgroup analyzes according to different stroke conditions and different anesthetic drugs due to limitations of baseline data and few studies reporting specific anesthetic regimens. Finally, there are differences in the actual implementation of specific anesthesia methods among multiple centers, and this irremovable bias may also have an impact on outcomes. In particular, different anesthesiologists and neurologists may have personal preferences, which cannot be eliminated.

## Conclusion

Utilizing IAT without GA presents clear benefits for patients suffering from ischemic stroke. These advantages include a reduced risk of mortality, an increased rate of successful reperfusion, and shorter hospital stays. Regarding neurological outcomes, patients undergoing IAT without GA tend to experience fewer short-term postoperative deficits. However, when considering long-term neurological outcomes, GA may yield superior results. In addition, GA may have a smaller risk of vascular perforation and a higher risk of intracranial hemorrhage after surgery. Therefore, for the anesthesia plan of IAT in the future, excluding unstable or critically dangerous patients, patients with high risk of aspiration pneumonia and other routine non-special situations, we can consider more non-intubation GA methods, such as MAC and CS. When conditions permit, the anesthesia plan should be fully evaluated and discussed by anesthesiologists and neurologists, and the decision should be made after the three parties have discussed and educated the patient’s family.

### Electronic supplementary material

Below is the link to the electronic supplementary material.

Supplementary Material 1

Supplementary Material 2

Supplementary Material 3

Supplementary Material 4

Supplementary Material 5

Supplementary Material 6

Supplementary Material 7

Supplementary Material 8

Supplementary Material 9

Supplementary Material 10

Supplementary Material 11

Supplementary Material 12

Supplementary Material 13

Supplementary Material 14

Supplementary Material 15

Supplementary Material 16

Supplementary Material 17

Supplementary Material 18

Supplementary Material 19

Supplementary Material 20

Supplementary Material 21

Supplementary Material 22

Supplementary Material 23

Supplementary Material 24

Supplementary Material 25

Supplementary Material 26

Supplementary Material 27

Supplementary Material 28

Supplementary Material 29

Supplementary Material 30

Supplementary Material 31

Supplementary Material 32

Supplementary Material 33

Supplementary Material 34

Supplementary Material 35

## Data Availability

All data generated or analysed during this study are included in this published article [and its supplementary information files].

## References

[1] Global regional (2019). National burden of neurological disorders, 1990–2016: a systematic analysis for the global burden of Disease Study 2016[J]. Lancet Neurol.

[2] Xu AD, Wang YJ, Wang DZ (2013). Consensus statement on the use of intravenous recombinant tissue plasminogen activator to treat acute ischemic stroke by the Chinese Stroke Therapy Expert Panel[J]. CNS Neurosci Ther.

[3] Powers WJ, Rabinstein AA, Ackerson T, Adeoye OM, Bambakidis NC, Becker K, Biller J, Brown M, Demaerschalk BM, Hoh B, Jauch EC, Kidwell CS, Leslie-Mazwi TM, Ovbiagele B, Scott PA, Sheth KN, Southerland AM, Summers DV (2019). Tirschwell D. L. Guidelines for the early management of patients with Acute ischemic stroke: 2019 update to the 2018 guidelines for the early management of Acute ischemic stroke: a Guideline for Healthcare professionals from the American Heart Association/American Stroke Association[J]. Stroke.

[4] Levy EI, Siddiqui AH, Crumlish A, Snyder KV, Hauck EF, Fiorella DJ, Hopkins LN, Mocco J (2009). First Food and Drug Administration-approved prospective trial of primary intracranial stenting for acute stroke: SARIS (stent-assisted recanalization in acute ischemic stroke)[J]. Stroke.

[5] Emberson J, Lees KR, Lyden P, Blackwell L, Albers G, Bluhmki E, Brott T, Cohen G, Davis S, Donnan G, Grotta J, Howard G, Kaste M, Koga M, von Kummer R, Lansberg M, Lindley RI, Murray G, Olivot JM, Parsons M, Tilley B, Toni D, Toyoda K, Wahlgren N, Wardlaw J, Whiteley W, del Zoppo GJ, Baigent C, Sandercock P, Hacke W (2014). Effect of treatment delay, age, and stroke severity on the effects of intravenous thrombolysis with alteplase for acute ischaemic stroke: a meta-analysis of individual patient data from randomised trials[J]. Lancet.

[6] Widimsky P, Snyder K, Sulzenko J, Hopkins LN, Stetkarova I (2023). Acute ischaemic stroke: recent advances in reperfusion treatment[J]. Eur Heart J.

[7] Berkhemer OA, Fransen PS, Beumer D, van den Berg LA, Lingsma HF, Yoo AJ, Schonewille WJ, Vos JA, Nederkoorn PJ, Wermer MJ, van Walderveen MA, Staals J, Hofmeijer J, van Oostayen JA, Lycklama à Nijeholt GJ, Boiten J, Brouwer PA, Emmer BJ, de Bruijn SF, van Dijk LC, Kappelle LJ, Lo RH, van Dijk EJ, de Vries J, de Kort PL, van Rooij WJ, van den Berg JS, van Hasselt BA, Aerden LA, Dallinga RJ, Visser MC, Bot JC, Vroomen PC, Eshghi O, Schreuder TH, Heijboer RJ, Keizer K, Tielbeek AV, den, Hertog HM, Gerrits DG, van den Berg-Vos RM, Karas GB, Steyerberg EW, Flach HZ, Marquering HA, Sprengers ME, Jenniskens SF, Beenen LF, van den Berg R, Koudstaal PJ, van Zwam WH, Roos Y. B., van der Lugt A, van Oostenbrugge RJ, Majoie CB. Dippel D. W. A randomized trial of intraarterial treatment for acute ischemic stroke[J]. N Engl J Med, 2015, 372(1): 11–20.

[8] Goyal M, Demchuk AM, Menon BK, Eesa M, Rempel JL, Thornton J, Roy D, Jovin TG, Willinsky RA, Sapkota BL, Dowlatshahi D, Frei DF, Kamal NR, Montanera WJ, Poppe AY, Ryckborst KJ, Silver FL, Shuaib A, Tampieri D, Williams D, Bang OY, Baxter BW, Burns PA, Choe H, Heo JH, Holmstedt CA, Jankowitz B, Kelly M, Linares G, Mandzia JL, Shankar J, Sohn SI, Swartz RH, Barber PA, Coutts SB, Smith EE, Morrish WF, Weill A, Subramaniam S, Mitha AP, Wong JH, Lowerison M. W., Sajobi T. T., Hill M. D. Randomized assessment of rapid endovascular treatment of ischemic stroke[J]. N Engl J Med, 2015, 372(11): 1019–1030. 10.1056/NEJMoa1414905 25671798

[9] Campbell BC, Mitchell PJ, Kleinig TJ, Dewey HM, Churilov L, Yassi N, Dowling YB, Parsons RJ, Oxley MW, Wu TJ, Brooks TY, Simpson M, Miteff MA, Levi F, Krause CR, Harrington M, Faulder TJ, Steinfort KC, Priglinger BS, Ang M, Scroop T, Barber R, McGuinness PA, Wijeratne B, Phan T, Chong TG, Chandra W, Bladin RV, Badve CF, Rice M, de Villiers H, Ma L, Desmond H, Donnan PM (2015). Davis S. M. Endovascular therapy for ischemic stroke with perfusion-imaging selection[J]. N Engl J Med.

[10] Campbell BCV, van Zwam WH, Goyal M, Menon BK, Dippel DWJ, Demchuk AM, Bracard S, White P, Dávalos A, Cblm M, van der Lugt A, Ford GA, de la Ossa NP, Kelly M, Bourcier R, Donnan GA, Ybwem R, Nogueira BOY, Devlin RG, van den Berg TG, Clarençon LA, Burns F, Carpenter P, Berkhemer J, Yavagal OA, Pereira DR, Ducrocq VM, Dixit X, Quesada A, Epstein H, Davis J, Jansen SM, Rubiera O, Urra M, Micard X, Lingsma E, Naggara HF, Brown O, Guillemin S, Muir F, van Oostenbrugge KW, Saver RJ, Jovin JL, Hill TG, Mitchell MD (2018). Effect of general anaesthesia on functional outcome in patients with anterior circulation ischaemic stroke having endovascular thrombectomy versus standard care: a meta-analysis of individual patient data[J]. Lancet Neurol.

[11] Schönenberger S, Uhlmann L, Hacke W, Schieber S, Mundiyanapurath S, Purrucker JC, Nagel S, Klose C, Pfaff J, Bendszus M, Ringleb PA, Kieser M, Möhlenbruch MA, Bösel J (2016). Effect of conscious sedation vs General Anesthesia on early neurological improvement among patients with ischemic stroke undergoing endovascular thrombectomy: a randomized clinical Trial[J]. JAMA.

[12] Simonsen CZ, Yoo AJ, Sørensen LH, Juul N, Johnsen SP, Andersen G, Rasmussen M (2018). Effect of General Anesthesia and Conscious Sedation during Endovascular Therapy on Infarct Growth and Clinical outcomes in Acute ischemic stroke: a randomized clinical Trial[J]. JAMA Neurol.

[13] Löwhagen Hendén P, Rentzos A, Karlsson JE, Rosengren L, Leiram B, Sundeman H, Dunker D, Schnabel K, Wikholm G, Hellström M, Ricksten SE (2017). General Anesthesia Versus Conscious Sedation for Endovascular Treatment of Acute ischemic stroke: the AnStroke Trial (Anesthesia during Stroke)[J]. Stroke.

[14] Schönenberger S, Hendén PL, Simonsen CZ, Uhlmann L, Klose C, Pfaff JAR, Yoo AJ, Sørensen LH, Ringleb PA, Wick W, Kieser M, Möhlenbruch MA, Rasmussen M, Rentzos A (2019). Bösel J. Association of General Anesthesia vs Procedural Sedation with Functional Outcome among patients with Acute ischemic stroke undergoing thrombectomy: a systematic review and Meta-analysis[J]. JAMA.

[15] Page MJ, Moher D, Bossuyt PM, Boutron I, Hoffmann TC, Mulrow CD, Shamseer L, Tetzlaff JM, Akl EA, Brennan SE, Chou R, Glanville J, Grimshaw JM, Hróbjartsson A, Lalu MM, Li T, Loder EW, Mayo-Wilson E, McDonald S, McGuinness LA, Stewart LA, Thomas J, Tricco AC, Welch VA, Whiting P, McKenzie JE (2021). PRISMA 2020 explanation and elaboration: updated guidance and exemplars for reporting systematic reviews[J]. BMJ.

[16] Higgins Julian PT. Green Sally. Cochrane handbook for systematic reviews of interventions[J], 2008,:.

[17] Abou-Chebl A, Zaidat OO, Castonguay AC, Gupta R, Sun CH, Martin CO, Holloway WE, Mueller-Kronast N, English JD, Linfante I, Dabus G, Malisch TW, Marden FA, Bozorgchami H, Xavier A, Rai AT, Froehler MT, Badruddin A, Nguyen TN, Taqi M, Abraham MG, Janardhan V, Shaltoni H, Novakovic R, Yoo AJ, Chen PR, Britz GW, Kaushal R, Nanda A, Issa MA, Nogueira RG (2014). North American SOLITAIRE Stent-Retriever Acute Stroke Registry: choice of anesthesia and outcomes[J]. Stroke.

[18] Bekelis K, Missios S, MacKenzie TA, Tjoumakaris S, Jabbour P (2017). Anesthesia technique and outcomes of mechanical thrombectomy in patients with Acute Ischemic Stroke[J]. Stroke.

[19] Berkhemer OA, van den Berg LA, Fransen PS, Beumer D, Yoo AJ, Lingsma HF, Schonewille WJ, van den Berg R, Wermer MJ, Boiten J, Lycklama À, Nijeholt GJ, Nederkoorn PJ, Hollmann MW, van Zwam WH, van der Lugt A, van Oostenbrugge RJ, Majoie CB, Dippel DW (2016). Roos Y. B. The effect of anesthetic management during intra-arterial therapy for acute stroke in MR CLEAN[J]. Neurology.

[20] Cappellari M, Pracucci G, Forlivesi S, Saia V, Nappini S, Nencini P, Inzitari D, Greco L, Sallustio F, Vallone S, Bigliardi G, Zini A, Pitrone A, Grillo F, Musolino R, Bracco S, Tinturini R, Tassi R, Bergui M, Cerrato P, Saletti A, De Vito A, Casetta I, Gasparotti R, Magoni M, Castellan L, Malfatto L, Menozzi R, Scoditti U, Causin F, Baracchini C, Puglielli E, Casalena A, Ruggiero M, Malatesta E, Comelli C, Chianale G, Lauretti DL, Mancuso M, Lafe E, Cavallini A, Cavasin N, Critelli A, Ciceri EFM, Bonetti B, Chiumarulo L, Petruzzelli M, Giorgianni A, Versino M, Ganimede MP, Tinelli A, Auteri W, Petrone A, Guidetti G, Nicolini E, Allegretti L, Tassinari T, Filauri P, Sacco S, Pavia M, Invernizzi P, Nuzzi NP, Carmela Spinelli M, Amistà P, Russo M, Ferrandi D, Corraine S, Craparo G, Mannino M, Simonetti L, Toni D, Mangiafico S. General Anesthesia Versus Conscious Sedation and Local Anesthesia During Thrombectomy for Acute Ischemic Stroke[J]. Stroke, 2020, 51(7): 2036–2044. 10.1161/STROKEAHA.120.028963 32517584

[21] Chabanne R, Geeraerts T, Begard M, Balança B, Rapido F, Degos V, Tavernier B, Molliex S, Velly L, Verdonk F, Lukaszewicz AC, Perrigault PF, Albucher JF, Cognard C, Guyot A, Fernandez C, Masgrau A, Moreno R, Ferrier A, Jaber S, Bazin JE, Pereira B (2023). Futier E. outcomes after Endovascular Therapy with Procedural Sedation vs General Anesthesia in patients with Acute ischemic stroke: the AMETIS randomized clinical Trial[J]. JAMA Neurol.

[22] Farag E, Liang C, Mascha EJ, Toth G, Argalious M, Manlapaz M, Gomes J, Ebrahim Z, Hussain MS (2022). Oxygen saturation and postoperative mortality in patients with Acute ischemic stroke treated by endovascular Thrombectomy[J]. Anesth Analg.

[23] Goldhoorn RB, Bernsen MLE, Hofmeijer J, Martens JM, Lingsma HF, Dippel DWJ, van der Lugt A, Wffa B, Ybwem R, Cblm M, Boiten VJA, Emmer J, van Oostenbrugge B, van Zwam RJ (2020). Investigators Mr Clean Registry. Anesthetic management during endovascular treatment of acute ischemic stroke in the MR CLEAN Registry[J]. Neurology.

[24] Hu G, Shi Z, Li B, Shao W, Xu B (2021). General anesthesia versus monitored anesthesia care during endovascular therapy for vertebrobasilar stroke[J]. Am J Transl Res.

[25] Jagani M, Brinjikji W, Rabinstein AA, Pasternak JJ, Kallmes DF (2016). Hemodynamics during anesthesia for intra-arterial therapy of acute ischemic stroke[J]. J Neurointerv Surg.

[26] Janssen H, Buchholz G, Killer M, Ertl L, Bruckmann H, Lutz J (2016). General Anesthesia Versus Conscious Sedation in Acute Stroke Treatment: the importance of Head Immobilization[J]. Cardiovasc Intervent Radiol.

[27] John S, Thebo U, Gomes J, Saqqur M, Farag E, Xu J, Wisco D, Uchino K, Hussain M (2014). Intra-arterial therapy for acute ischemic stroke under general anesthesia versus monitored anesthesia care[J]. Cerebrovasc Dis.

[28] Just C, Rizek P, Tryphonopoulos P, Pelz D, Arango M (2016). Outcomes of General Anesthesia and Conscious Sedation in Endovascular Treatment for Stroke[J]. Can J Neurol Sci.

[29] Li F, Deshaies EM, Singla A, Villwock MR, Melnyk V, Gorji R, Yang ZJ (2014). Impact of anesthesia on mortality during endovascular clot removal for acute ischemic stroke[J]. J Neurosurg Anesthesiol.

[30] Li Z, Ma H, Li B, Zhang L, Zhang Y, Xing P, Zhang Y, Zhang X, Zhou Y, Huang Q, Li Q, Zuo Q, Ye X, Liu J, Qureshi AI, Chen W, Yang P (2023). Investigators Direct-Mt. Impact of anesthesia modalities on functional outcome of mechanical thrombectomy in patients with acute ischemic stroke: a subgroup analysis of DIRECT-MT trial[J]. Eur J Med Res.

[31] Liang F, Wu Y, Wang X, Yan L, Zhang S, Jian M, Liu H, Wang A, Wang F, Han R (2023). Group Canvas Ii. General Anesthesia vs conscious sedation for endovascular treatment in patients with posterior circulation Acute ischemic stroke: an exploratory Randomized Clinical Trial[J]. JAMA Neurol.

[32] Maurice A, Eugene F, Ronziere T, Devys JM, Taylor G, Subileau A, Huet O, Gherbi H, Laffon M, Esvan M, Laviolle B, Beloeil H (2022). Group Gass Study, the French Society of Anesthesiologists Research Network. General Anesthesia versus Sedation, both with Hemodynamic Control, during Intraarterial Treatment for Stroke: the GASS Randomized Trial[J]. Anesthesiology.

[33] Mundiyanapurath S, Schonenberger S, Rosales ML, Carrilho Romeiro AM, Mohlenbruch M, Bendszus M, Hacke W, Bosel J (2015). Circulatory and respiratory parameters during Acute Endovascular Stroke Therapy in Conscious Sedation or General Anesthesia[J]. J Stroke Cerebrovasc Dis.

[34] Peng Y, Wu Y, Huo X, Wu P, Zhou Y, Li J, Liang F, Liu X, Pan Y, Miao Z, Han R (2019). Endovascular therapy for Acute ischemic stroke trial group. Outcomes of Anesthesia Selection in Endovascular Treatment of Acute Ischemic Stroke[J]. J Neurosurg Anesthesiol.

[35] Pop Raoul S, François HN, Emmanuel H, Oana M, Ioan M, Dan M, Monica S, Mihaela C, Salvatore W, Valérie G, Roxana B, Serge. Anxionnat René, Richard Sébastien, Beaujeux Rémy, Gory Benjamin. Local anesthesia versus general anesthesia during endovascular therapy for acute stroke: a propensity score analysis[J]. Journal of NeuroInterventional Surgery, 2021, 13(3): 207–211. 10.1136/neurintsurg-2020-015916 32487768

[36] Ren C, Xu G, Liu Y, Liu G, Wang J, Gao J (2020). Effect of conscious sedation vs. General Anesthesia on outcomes in patients undergoing mechanical thrombectomy for Acute ischemic stroke: a prospective Randomized Clinical Trial[J]. Front Neurol.

[37] van den Berg Lucie A, Koelman Diederik LH, Berkhemer Olvert A, Rozeman Anouk D, Fransen Puck SS, Debbie B, Dippel Diederik W, van der Lugt Aad J, van Zwam Wim H, Brouwer Patrick A, Sjoerd J, Boiten Jelis, Lycklama à Nijeholt Geert A., Vos Jan Albert, Schonewille Wouter J., Majoie Charles B. L. M., Roos Yvo B. W. E. M., de Bruijn Sebastiaan, van Lukas D. Kappelle Jaap, Lo Rob, de Kort Paul, van Rooij Willem Jan, Hofmeijer Jeannette, van Oostayen Jacques, van Dijk Ewoud, de Vries Joost, Schreuder Tobien, Heijboer Roel, Vroomen Patrick, Eshghi Omid, Aerden Leo, Dallinga René, van den Berg Jan, van Hasselt Boudewijn, den Hertog Heleen, Tielbeek Alexander, Wermer Marieke, van Walderveen Marianne. Type of Anesthesia and Differences in Clinical Outcome After Intra-Arterial Treatment for Ischemic Stroke[J]. Stroke, 2015, 46(5): 1257–1262. 10.1161/STROKEAHA.115.008699 25851766

[38] Sun J, Liang F, Wu Y, Zhao Y, Miao Z, Zhang L, Gelb AW, Chan MTV, Peng Y, Han R (2020). Investigators Canvas Pilot Trial. Choice of ANesthesia for EndoVAScular Treatment of Acute ischemic stroke (CANVAS): results of the CANVAS Pilot randomized controlled Trial[J]. J Neurosurg Anesthesiol.

[39] Wagner B, Lorscheider J, Wiencierz A, Blackham K, Psychogios M, Bolliger D, De Marchis GM, Engelter ST, Lyrer P, Wright PR, Fischer U, Mordasini P, Nannoni S, Puccinelli F, Kahles T, Bianco G, Carrera E, Luft AR, Cereda CW, Kagi G, Weber J, Nedeltchev K, Michel P, Gralla J, Arnold M, Bonati LH (2022). Swiss stroke Registry investigators. Endovascular treatment for Acute Ischemic Stroke with or without General Anesthesia: a matched Comparison[J]. Stroke.

[40] Wu L, Jadhav AP, Chen J, Sun C, Ji K, Li W, Zhao W, Li C, Wu C, Wu D, Ji X (2020). Local anesthesia vs general anesthesia during endovascular therapy for acute posterior circulation stroke[J]. J Neurol Sci.

[41] Wu L, Jadhav AP, Zhao W, Wu D, Chen J, Yang S, Wu C, Li C, Duan J, Ding Y, Ji X (2019). General anesthesia vs local anesthesia during mechanical thrombectomy in acute ischemic stroke[J]. J Neurol Sci.

[42] Weyland CS, Chen M, Potreck A, Jäger LB, Seker F, Schönenberger S, Bendszus M (2021). Möhlenbruch M. Sedation Mode during Endovascular Stroke Treatment in the posterior Circulation-Is conscious sedation for eligible patients feasible?[J]. Front Neurol.

[43] Terceño M, Silva Y, Bashir S, Vera-Monge VA, Cardona P, Molina C, Chamorro Á, de la Ossa NP, Hernández-Pérez M, Werner M, Camps-Renom P, Rodríguez-Campello A, Cánovas D, Purroy F, Serena J (2021). Impact of general anesthesia on posterior circulation large vessel occlusions after endovascular thrombectomy[J]. Int J Stroke.

[44] Du H, Tong X, Sun X, Shi Z, Liu B, Gao F, Miao Z, Zhang D (2020). Effect of anesthesia strategy during endovascular therapy on 90-day outcomes in acute basilar artery occlusion: a retrospective observational study[J]. BMC Neurol.

[45] Jadhav AP, Bouslama M, Aghaebrahim A, Rebello LC, Starr MT, Haussen DC, Ranginani M, Whalin MK, Jovin TG, Nogueira RG (2017). Monitored Anesthesia Care vs Intubation for Vertebrobasilar Stroke Endovascular Therapy[J]. JAMA Neurol.

[46] Li F, Wan J, Song J, Yuan J, Kong W, Huang J, Luo W, Wu D, Li L, Chen L, Zhao C, Chen J, Tao H, Sang H, Qiu Z, Zi W, Yang Q, Chen X, Li H, Peng F (2022). Impact of anesthetic strategy on outcomes for patients with acute basilar artery occlusion undergoing mechanical thrombectomy[J]. J Neurointerv Surg.

[47] Werner C (2010). Neuroprotection in acute cerebral ischemia: can we improve clinical outcomes?[J]. Best Pract Res Clin Anaesthesiol.

[48] Chiao SS, Zuo Z (2019). General anesthetics are Neuroprotective[J]. J Neurosurg Anesthesiol.

[49] Finks JF, Osborne NH, Birkmeyer JD (2011). Trends in hospital volume and operative mortality for high-risk surgery[J]. N Engl J Med.

[50] Walsh CT, Propofol (2018). Milk of Amnesia[J]. Cell.

[51] Zanos P, Moaddel R, Morris PJ, Riggs LM, Highland JN, Georgiou P, Pereira EFR, Albuquerque EX, Thomas CJ, Gould ZCA (2018). Ketamine and ketamine metabolite pharmacology: insights into therapeutic Mechanisms[J]. Pharmacol Rev.

[52] Xue Y, Wang AZ (2020). DJ-1 plays a neuroprotective role in SH-SY5Y cells by modulating Nrf2 signaling in response to lidocaine-mediated oxidative stress and apoptosis[J]. Kaohsiung J Med Sci.

