# Supplementary material for: Comparison of anesthesia methods for intra-arterial therapy of patients with acute ischemic stroke: an updated meta-analysis and systematic review
Source: BMC Anesthesiol. 2024 Jul 18;24:243. doi: 10.1186/s12871-024-02633-3 (PMC11256490; doi:10.1186/s12871-024-02633-3)
Supplement: Supplementary file 1 — Supplementary Material 1 [file 12871_2024_2633_MOESM1_ESM.docx]

**Identification of studies via databases and registers**

Records identified from:

PubMed (n = 346)

WOS (n = 501)

Scopus (n = 142)

The Cochrane library (n = 230)

Records removed *before screening*:

Duplicate records removed (n = 114)

**Identification**

Records screened

(n = 1105)

Records excluded

(n = 1064)

Reports sought for retrieval

(n = 41)

**Screening**

Reports excluded:

Not original research (n = 3)

No comparison between GA and non-GA (n = 6)

Data cannot be converted into a usable form (n = 5)

Studies included in review

(n = 27)

**Included**
