# Supplementary material for: Comparison of anesthesia methods for intra-arterial therapy of patients with acute ischemic stroke: an updated meta-analysis and systematic review
Source: BMC Anesthesiol. 2024 Jul 18;24:243. doi: 10.1186/s12871-024-02633-3 (PMC11256490; doi:10.1186/s12871-024-02633-3)
Supplement: Supplementary file 27 — Supplementary Material 27 [file 12871_2024_2633_MOESM27_ESM.docx]

#1 ((("Anesthesia, General"[Mesh]) OR (Anesthesias, General[Title/Abstract])) OR (General Anesthesia[Title/Abstract])) OR (General Anesthesias[Title/Abstract])

#2 (Monitored anesthesia care[Title/Abstract]) OR (MAC[Title/Abstract])

#3 ((("Conscious Sedation"[Mesh]) OR (Sedation, Moderate[Title/Abstract])) OR (Moderate Sedation[Title/Abstract])) OR (Sedation, Conscious[Title/Abstract])

#4 ((((("Anesthesia, Local"[Mesh]) OR (Local Anesthesia[Title/Abstract])) OR (Anesthesia, Infiltration[Title/Abstract])) OR (Infiltration Anesthesia[Title/Abstract])) OR (Neural Therapy of Huneke[Title/Abstract])) OR (Huneke Neural Therapy[Title/Abstract])

#5 (((((((((((((((((((((((((((("Stroke"[Mesh]) OR (Strokes[Title/Abstract])) OR (Cerebrovascular Accident[Title/Abstract])) OR (Cerebrovascular Accidents[Title/Abstract])) OR (CVA (Cerebrovascular Accident[Title/Abstract]))) OR (CVAs (Cerebrovascular Accident[Title/Abstract]))) OR (Cerebrovascular Apoplexy[Title/Abstract])) OR (Apoplexy, Cerebrovascular[Title/Abstract])) OR (Vascular Accident, Brain[Title/Abstract])) OR (Brain Vascular Accident[Title/Abstract])) OR (Brain Vascular Accidents[Title/Abstract])) OR (Vascular Accidents, Brain[Title/Abstract])) OR (Cerebrovascular Stroke[Title/Abstract])) OR (Cerebrovascular Strokes[Title/Abstract])) OR (Stroke, Cerebrovascular[Title/Abstract])) OR (Strokes, Cerebrovascular[Title/Abstract])) OR (Apoplexy[Title/Abstract])) OR (Cerebral Stroke[Title/Abstract])) OR (Cerebral Strokes[Title/Abstract])) OR (Stroke, Cerebral[Title/Abstract])) OR (Strokes, Cerebral[Title/Abstract])) OR (Stroke, Acute[Title/Abstract])) OR (Acute Stroke[Title/Abstract])) OR (Acute Strokes[Title/Abstract])) OR (Strokes, Acute[Title/Abstract])) OR (Cerebrovascular Accident, Acute[Title/Abstract])) OR (Acute Cerebrovascular Accident[Title/Abstract])) OR (Acute Cerebrovascular Accidents[Title/Abstract])) OR (Cerebrovascular Accidents, Acute[Title/Abstract])

#6 #2 OR #3

#7 #4 OR #6

#8 #1 AND #7

#9 #5 AND #8
