# Supplementary material for: Comparison of anesthesia methods for intra-arterial therapy of patients with acute ischemic stroke: an updated meta-analysis and systematic review
Source: BMC Anesthesiol. 2024 Jul 18;24:243. doi: 10.1186/s12871-024-02633-3 (PMC11256490; doi:10.1186/s12871-024-02633-3)
Supplement: Supplementary file 35 — Supplementary Material 35 [file 12871_2024_2633_MOESM35_ESM.docx]

| Reference | Selection | Comparability | Outcome | Score |
| --- | --- | --- | --- | --- |
| Abou 2014 | ★★★★ | ★ | ★★ | 7 |
| Bekelis 2017 | ★★★★ | ★★ | ★★ | 8 |
| Cappellari 2020 | ★★★★ | ★★ | ★★★ | 9 |
| Farag 2022 | ★★★★ | ★★ | ★★ | 8 |
| Hu 2021 | ★★★★ | ★★ | ★★★ | 9 |
| Jagani 2015 | ★★★ | ★★ | ★★ | 7 |
| Janssen 2016 | ★★★ | ★★ | ★★ | 7 |
| John 2014 | ★★★★ | ★★ | ★★ | 8 |
| Just 2016 | ★★★★ | ★ | ★★ | 7 |
| Li 2016 | ★★★★ | ★★ | ★★ | 8 |
| Pop 2021 | ★★★★ | ★★ | ★★★ | 9 |
| Vandenberg 2015 | ★★★ | ★★ | ★★★ | 8 |
| Wagner 2022 | ★★★ | ★★ | ★★★ | 8 |
| Wu 2020 | ★★★ | ★★ | ★★★ | 8 |

Table 3. Results of NOS quality assessment.
